# Short-Term Reproducibility of MUC5AC Measurement in Human Tear Fluid

**DOI:** 10.3390/diagnostics11010057

**Published:** 2021-01-02

**Authors:** Ashley M. Woodward, Michelle Senchyna, Pablo Argüeso

**Affiliations:** 1 Department of Ophthalmology, Schepens Eye Research Institute of Massachusetts Eye and Ear, Harvard Medical School, Boston, MA 02114, USA; ashley_woodward@meei.harvard.edu; 2 Allergan Plc, Irvine, CA 92612, USA; msenchyna@aeriepharma.com

**Keywords:** biomarker, MUC5AC, repetitive sample collection, short-term reproducibility, human tear fluid

## Abstract

The assessment of tear fluid components is a common and valuable approach to understanding ocular surface disease and testing the efficacy of novel therapeutic strategies. However, the interpretation and utility of the findings can be limited by changes in the composition of the tear film, particularly in studies requiring repetitive patient sampling. Here, tear samples were collected twice within a one-hour interval to evaluate the short-term reproducibility of an immunoassay aimed to measure the amount of MUC5AC mucin. We found no statistical difference in total protein or MUC5AC content between the two consecutive collections of tear fluid, although the inter-individual variability in each group was high, with coefficients of variation exceeding 30% and 50%, respectively. Scatterplots showed a significant correlation in both protein and MUC5AC following collection within a one-hour interval. These data indicate that, regardless of the high inter-individual variability, repeated collection of tear fluid within an hour interval produces reproducible intra-individual data in terms of MUC5AC mucin content, and suggest that the normal mucin composition of the tear fluid can be re-established within an hour of the initial collection.

## 1. Introduction

The human tear film is a multifunctional fluid secreted to protect the ocular surface from injury and to provide a smooth refractive surface for optimal visual acuity. It is composed of a complex mixture of electrolytes, lipids, mucins, and a large variety of proteins, peptides and glycoproteins [1]. The fluid itself is mainly produced by the lacrimal gland and in minor amounts by the accessory glands of Wolfring and Krause [2]. Conjunctival goblet cells produce and secrete the large gel-forming mucin MUC5AC, whereas the meibomian glands of the lid margin are mainly responsible for producing a mixture of various polar and nonpolar lipids. Disturbed tear film composition has been found in many ocular surface diseases such as dry eye [3].

Developing reproducible tests for the quantitative and objective assessment of tear fluid production and composition is critical to detect changes in ocular surface disease and for the successful development of therapeutic strategies [4,5,6]. The use of standard biological detection methods with low sensitivity have hampered these studies in the past. However, recent advances and developments in instrumentation now allow the analysis of many tear components at high sensitivity using samples from individual subjects [7]. Having a good understanding of the potential changes that may occur to the tear film during the repetitive collection of tear fluid is important in the interpretation of these studies. This is particularly relevant in studies such as those evaluating temporal changes in tear composition following therapy or investigating disease biomarkers. The current study was carried out to assess the inter-individual variability and intra-individual reproducibility of MUC5AC levels in samples of tear fluid collected within a one-hour interval.

## 2. Materials and Methods

### 2.1. Antibodies and Reagents

Human MCF7 (breast cancer) whole-cell lysate, mouse anti-human MUC5AC (CLH2), and goat anti-mouse IgG-HRP were purchased from Santa Cruz Biotechnology (Dallas, TX, USA). Human stomach whole-cell lysate and Sialidase A were from Abcam (Cambridge, MA, USA) and Prozyme (Hayward, CA, USA), respectively. The Micro BCA Protein Assay Kit and the SuperSignal West Pico chemiluminescent substrate were obtained from Thermo Scientific (Rockford, IL, USA). Molecular biology grade agarose was purchased from Research Products International (Mount Prospect, IL, USA).

### 2.2. Subjects

This study was IRB approved and the protocol adhered to the tenets of the Declaration of Helsinki. All of the subjects gave their informed consent prior to participation in this study. All subjects were enrolled at one site (South San Francisco) and the study was conducted in a single visit not aiming to treat an underlying condition. Subjects were excluded from the study if they were less than 18 years of age, had uncontrolled ocular disease or systemic disease, wore contact lenses within 24 h of the study visit or used any topical ocular medications (with the exception of artificial tears) within 7 days of the study visit. The use of artificial tears was not permitted during the study visit.

A total of 20 subjects participated in this study (six female and 14 male), with 17 providing tear fluid samples of sufficient volume (at least 5 μL) during three sequential collections. Only samples from these 17 subjects were analyzed. The mean age of all enrolled subjects was 38.3 ± 13.8 years and the mean Ocular Surface Disease Index (OSDI) score was 7.0 ± 9.6 (range: 0 to 41). The mean age of the 17 subjects that provided sufficient tears for analysis was 39.5 ± 14.7 years and the mean OSDI score was 7.1 ± 10.4 (range: 0 to 42).

### 2.3. Tear Sample Collection

Tear samples were collected from both eyes a total of three times by the same investigator during the study visit. For each collection, the fluid from the left and right eyes was combined. Basal tears were collected twice, separated by approximately 60 min, using disposable 5 μL graduated glass microcapillary tubes gently placed just above the lower temporal tear meniscus, minimizing contact of the tip with the eye surface to avoid reflex tears. Basal tear collection in each eye was performed until a maximum of 5 μL was collected or 5 min had elapsed. After the second basal tear collection, the surface of the eye was gently washed by pipetting 50 μL of sterile saline (0.90% sodium chloride; Unisol 4, Alcon) into the inferior fornix using a sterile transfer pipet. The subjects were asked to briefly move their eyes to mix the tear fluid content. The tear fluid from each subject was then immediately collected using the same sterile transfer pipette. All samples were immediately placed in small tubes, centrifuged to remove cellular debris, and stored at −80 °C until analysis.

### 2.4. Protein Analysis and Immunoblotting

Protein concentration was determined with a protein assay reagent kit (MicroBCA; Pierce, Rockford, IL), using a dilution series of bovine serum albumin as the standard. For analysis of MUC5AC, samples were resolved by agarose gel electrophoresis (1%, *wt*/*vol*) and transferred onto nitrocellulose membranes (Bio-Rad, Hercules, CA, USA) by vacuum blotting as described previously [8]. Nonspecific binding to the membranes was blocked by incubation with 5% (*wt*/*vol*) nonfat dry milk in Tris-buffered saline containing 0.1% Tween-20 (T-TBS) for 1 h at room temperature. Membranes were then incubated with mouse anti-MUC5AC (1:100) in 5% nonfat dry milk in T-TBS overnight at 4 °C, followed by the corresponding peroxidase-conjugated anti-mouse IgG (1:5000) for 1 h at room temperature. Peroxidase activity was detected using the SuperSignal West Pico chemiluminescent substrate and imaged using the gel documentation system G:BOX (Syngene, Frederick, MD, USA). Band intensities were quantified by densitometry (ImageJ Software; National Institutes of Health, Bethesda, MD; in the public domain, available at http://rsbweb.nih.gov/ij/).

### 2.5. Sialidase A Treatment

Tear samples (4 μL) were treated with 10 mU Sialidase A from *Arthrobacter ureafaciens* (Prozyme; Hayward, CA, USA), which cleaves α2-3/6/8/9-linked sialic acid. Enzymatic treatments were carried out for 1 h at 37 °C in 50 mM sodium phosphate, pH 6.0, as previously described (Argueso and Sumiyoshi, 2006).

### 2.6. Statistical Analysis

Statistical analysis was carried out with GraphPad Prism 7 (GraphPad Software, San Diego, CA, USA) for Macintosh. All data are shown as Mean ± SD. The mean values from each group were compared using one-way analysis of variance (ANOVA) with Tukey’s post hoc test. In addition, mean change data between basal collections are presented along with the 95% confidence intervals. In all tests, *p* values < 0.05 were considered statistically significant.

## 3. Results and Discussion

**Figure 1:**
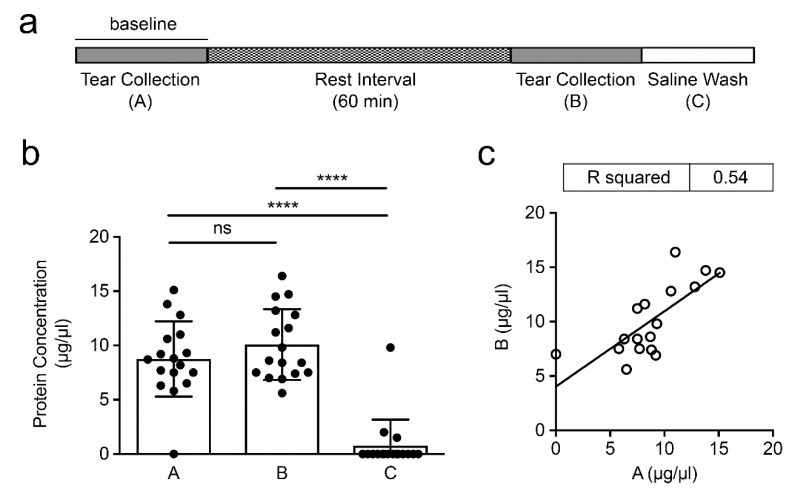
Analysis of total protein content and correlation between consecutive tear fluid collections. (**a**) Timeline showing the experimental steps of this study. (**b**) Protein concentrations of consecutive tear fluid collections (A, baseline; B, collection after rest interval; C, saline wash control). (**c**) Scatterplot depicting the correlation between (A) and (B). Symbols represent experimental data and lines represent regression fit. Data are shown as mean ± SD. Significance in (**b**) was determined using one-way ANOVA with Tukey’s post hoc test. **** *p* < 0.0001; ns, nonsignificant.

Understanding the changes that might occur in the tear film during repetitive collection is important in order to ensure the correct interpretation of data collected in studies aimed at characterizing disease pathology or testing the efficacy of novel therapeutic strategies. To address whether the levels of MUC5AC are altered in the short term following the sampling of the ocular surface, we collected tear fluid before and after a resting interval of 60 min and determined the inter-individual variability and intra-individual reproducibility of the procedure. In these experiments, we washed the eyes with saline to investigate whether any residual material remained at the ocular surface after collection by microcapillary tube (Figure 1a).

We found that the total protein concentration during the initial collection was 8.8 ± 3.5 µg/μL (Figure 1b), in line with what has been previously reported for human tears [9,10]. We also observed a slight increase in protein content in the collection that followed the resting period,10.1 ± 3.3 µg/μL, although the difference was not statistically significant. These results were in line with previous studies showing that the protein concentration of the tear film remains relatively constant after repetitive collection during the day, and up to 7 days after the initial collection [11,12,13,14]. Further analysis of the data within the same individual revealed a significant correlation in protein content between the two consecutive short-term tear fluid collections (R^2^ = 0.54, *p* < 0.001), indicating that the intra-individual reproducibility of the procedure was consistent (Figure 1c). The inter-individual variability in each group, however, was high, with coefficients of variation exceeding 30%. This was not entirely surprising as there are genetic and environmental factors that can individually affect tear production and composition, such as hormonal and nutritional stimuli [15,16]. In these experiments, the saline wash group contained little or no protein, indicating that collection by microcapillary tube efficiently removes the protein content from the tear film.

**Figure 2:**
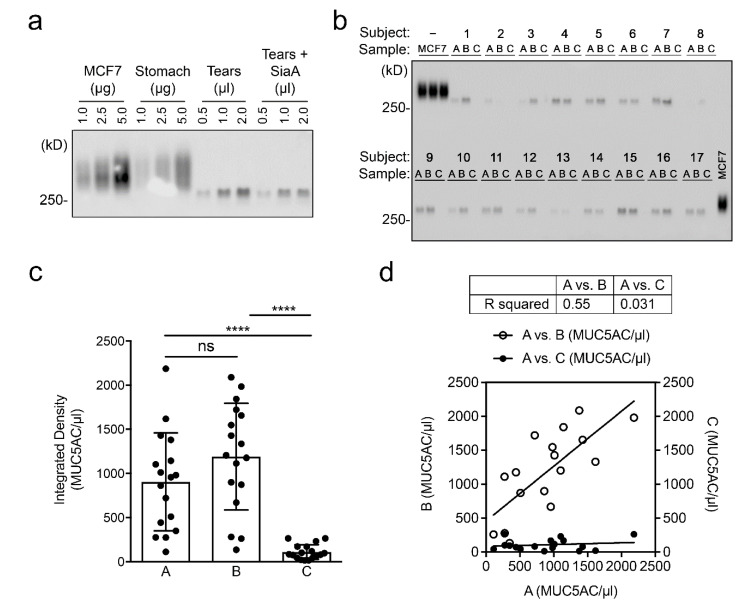
Analysis of MUC5AC mucin and correlation between consecutive tear fluid collections. (**a**) Detection of MUC5AC in pooled tear samples by immunoblotting. MCF7 whole-cell lysates and stomach mucin were used as positive controls. SiaA, Sialidase A. (**b**) Detection of MUC5AC in the 17 subjects that provided sufficient tears for analysis (A, baseline; B, collection after rest interval; C, saline wash control). (**c**) Quantification of the relative levels of MUC5AC per μL of tear fluid. (**d**) Scatterplot depicting the correlation between A vs. B or A vs. C. Symbols represent experimental data and lines represent regression fit. Data are shown as mean ± SD. Significance in (**c**) was determined using one-way ANOVA with Tukey’s post hoc test. **** *p* < 0.0001; ns, nonsignificant.

There are a number of tear proteins whose alteration is commonly indicative of ocular surface disease [7]. MUC5AC remains a therapeutic target for diseases associated with aberrant mucin expression such as dry eye [17]. Validating the reproducibility of assays to measure MUC5AC is therefore critical for monitoring disease activity and treatment efficacy. Here, we used an established immunoassay for detecting MUC5AC in human tear fluid. In this assay, tear proteins are separated by agarose gel electrophoresis, transferred to nitrocellulose membrane by vacuum blotting and the MUC5AC content probed with the CLH2 antibody [8]. We first evaluated the sensitivity of the assay using pooled tear fluid samples. As shown in Figure 2a, MUC5AC could be detected in the high-molecular-weight region of the gel (>250 kD), using as little as 0.5 µL of tear fluid, and was clearly noticeable using 2 µL. Pretreatment of the tear fluid with sialidase A to enhance CLH2 antibody reactivity towards the tandem repeat region of MUC5AC did not have a visible effect. Consequently, further experiments were performed using 2 µL of tear fluid without enzymatic digestion.

We compared the levels of MUC5AC between the two consecutive samplings of tear fluid. Analysis of the gel revealed no alteration in the electrophoretic migration of MUC5AC (Figure 2b), indicating that the mucin maintains its integrity during the resting period. Similar to the protein data, we found no statistical difference in the levels of MUC5AC between the two consecutive collections (Figure 2c). The results provided evidence supporting a good intra-individual reproducibility as shown by the significant correlation in MUC5AC levels (R^2^ = 0.55, *p* < 0.001) (Figure 2d). By comparison, no correlation between variables was observed in the saline wash control group (R^2^ = 0.031, *p* = 0.5). In these experiments we identified a high variability in the levels of MUC5AC among different individuals, with coefficients of variation greater than 50%. Previous studies have shown that the inter-individual variability appears to be dependent of the specific component being analyzed in the tear film and its secretion rate [12,18]. In this regard, MUC5AC is produced by goblet cells in the conjunctiva but the mechanisms regulating its secretion remain largely unknown. This study indicates that the levels of MUC5AC in the tear fluid can be re-established within an hour of the initial collection and should contribute to the standardization of protocols aimed to measure MUC5AC in the human tear film.

## Data Availability

The data presented in this study are available on request from the corresponding author.

## Abbreviations

ANOVA	One-way analysis of variance
kD	Kilodalton
MUC5AC	Mucin 5AC
OSDI	Ocular surface disease index
SiaA	Sialidase A
T-TBS	Tis-buffered saline containing 0.1% Tween-20

## References

[1] Ohashi Y., Dogru M., Tsubota K. (2006). Laboratory findings in tear fluid analysis. Clin. Chim. Acta.

[2] Tiffany J.M. (2008). The Normal Tear Film. Dev. Ophthalmol..

[3] Johnson E., Murphy M.P.J. (2004). Changes in the tear film and ocular surface from dry eye syndrome. Prog. Retin. Eye Res..

[4] Senchyna M., Wax M.B. (2008). Quantitative assessment of tear production: A review of methods and utility in dry eye drug discovery. J. Ocul. Biol. Dis. Inform..

[5] Sweeney D.F., Millar T.J., Raju S.R. (2013). Tear film stability: A review. Exp. Eye Res..

[6] Napoli P.E., Nioi M., D’Aloja E., Fossarello M. (2019). The Bull’s Eye Pattern of the Tear Film in Humans during Visual Fixation on En-Face Optical Coherence Tomography. Sci. Rep..

[7] Jacob J.T., Ham B. (2008). Compositional profiling and biomarker identification of the tear film. Ocul. Surf..

[8] Spurr-Michaud S., Argüeso P., Gipson I.K. (2007). Assay of mucins in human tear fluid. Exp. Eye Res..

[9] Posa A., Bräuer L., Schicht M., Garreis F., Beileke S., Paulsen F. (2013). Schirmer strip vs. capillary tube method: Non-invasive methods of obtaining proteins from tear fluid. Ann. Anat.—Anat. Anz..

[10] Ablamowicz A.F., Nichols J.J. (2017). Concentrations of MUC16 and MUC5AC using three tear collection methods. Mol. Vis..

[11] Huth S.W., Miller M.J., Leopold I.H. (1981). Calcium and Protein in Tears. Arch. Ophthalmol..

[12] Ng V., Cho P., Wong F., Chan Y. (2001). Variability of tear protein levels in normal young adults: diurnal (daytime) variation. Graefe’s Arch. Clin. Exp. Ophthalmol..

[13] Ng V., Cho P., Mak S., Lee A. (2000). Variability of tear protein levels in normal young adults: between-day variation. Graefe’s Arch. Clin. Exp. Ophthalmol..

[14] González N., Iloro I., Durán J.A., Elortza F., Suarez T. (2012). Evaluation of inter-day and inter-individual variability of tear peptide/protein profiles by MALDI-TOF MS analyses. Mol. Vis..

[15] Versura P., Fresina M., Campos E.C. (2007). Ocular surface changes over the menstrual cycle in women with and without dry eye. Gynecol. Endocrinol..

[16] Aragona P., Rania L., Micali A., Puzzolo D. (2013). Nutrition and Dry Eye. Curr. Ophthalmol. Rep..

[17] Argüeso P., Balaram M., Spurr-Michaud S., Keutmann H.T., Dana M.R., Gipson I.K. (2002). Decreased levels of the goblet cell mucin MUC5AC in tears of patients with Sjögren syndrome. Investig. Ophthalmol. Vis. Sci..

[18] Dammeier S., Martus P., Klose F., Seid M., Bosch D., D‘Alvise J., Ziemssen F., Dimopoulos S., Ueffing M. (2018). Combined Targeted Analysis of Metabolites and Proteins in Tear Fluid With Regard to Clinical Applications. Transl. Vis. Sci. Technol..

